# A Longitudinal Investigation of the Prevalence and Incidence of Self-Reported COVID-19 Disease and the Pandemic’s Impact Among Seventh-day Adventist and Non-Adventists Living in the UK

**DOI:** 10.1007/s10943-024-02129-x

**Published:** 2024-09-16

**Authors:** Robert K. Janko, Irmgard Haussmann, Ashok Patel

**Affiliations:** Department of Health Sciences, Birmingham City University, City South Campus, Seacole Building, Westbourne Rd, Birmingham, B15 3TN UK

**Keywords:** Diet, Nutrition, Infection, COVID-19, Adventist

## Abstract

This research investigated the prevalence and incidence of COVID-19 infection among Seventh-day Adventist Christians in the UK compared to non-Adventists and assessed the pandemic’s impact on their health and dietary intake. Seventh-day Adventists and non-Adventists in the UK completed an online survey, including a Food Frequency Questionnaire, a 24-h dietary recall, and health and lifestyle questions. Participants were followed for 2 years to determine COVID-19 incidence rates. The baseline survey was completed by 170 people, 86 of whom were Adventists. The follow-up at 2 years showed a significantly lower self-reported COVID-19 incidence among Adventists (OR 0.45, 95% CI 0.2, 1.0, p = 0.05). The incidence rate among Adventists was 65.48 per 1000 person-years versus 121.79 per 1000 person-years among non-Adventists. Adventists were less likely to experience long COVID (OR 0.30, 95% CI 0.12, 0.78, p = 0.01). Despite being older, Adventists had a significantly lower COVID-19 incidence rate. The Adventist lifestyle, including healthy eating habits, fasting, a plant-based diet, and abstaining from alcohol and coffee, was prevalent in this sample. More research is needed to explore the association between the Adventist lifestyle and infectious disease.

## Introduction

Seventh-day Adventist are encouraged to eat a healthy diet preferably vegetarian, but meats deemed “clean” based on the biblical criteria are allowed to be consumed (To et al., 2022). The presence of well-defined lifestyle practises generally followed by most church members such as abstinence from smoking or alcohol makes investigating Seventh-day Adventist populations desirable.

Surprisingly, there are not any published studies that have assessed the health and lifestyle of Adventists living in the UK. This also means that there are no publications examining the COVID-19 pandemic’s impact on Adventists living in the UK. The unexpected occurrence of the COVID-19 pandemic and the lockdowns imposed by governments around the world in response to the COVID-19 pandemic led to disruptions in food supplies and caused major issues with food availability and accessibility (Jafri et al., 2021) and had a significant impact on people's eating habits and mental health.

The study’s main aim was to examine the prevalence and incidence of COVID-19 infection due to the potentially protective nature of the Adventist lifestyle. Furthermore, most of the published studies that report positive results of Adventists are in the context of chronic diseases. Data regarding the prevalence of infectious diseases among Adventists are scarce; therefore, it was the objective of this study to explore the potential association between the risk of COVID-19 and the Adventist lifestyle. The study also assessed whether the government restrictions associated with COVID-19, which resulted in church closures around the UK for over a year, have affected the spiritual lives and practises of Adventists in a more significant way compared to non-religious people, and whether Adventists were more or less likely to report changes to their eating and lifestyle habits during the pandemic.

Kahleova & Barnard (2023) published an analysis from data gathered during the H1N1 pandemic between 1918 and 1919 at Adventist Sanitariums, which revealed that among the 446 Adventists who were treated at 10 different Adventist Sanitariums, the mortality rate was only 1.3%, which was 5 times lower than that in the US Army. Although mortality in both of the previously mentioned groups was lower than in the general US population, it is also worth noting that Adventist Sanitariums at the time utilised a plant-based diet as part of their treatment schedule.

A more recent study (Büssing et al., 2022) investigated the pandemic’s impact on Adventists’ attitudes and behaviours and showed that participants’ well-being scores remained stable during the pandemic and no negative effects of the pandemic on participants’ faith were found.

## Methods

Initial data collection for this study began in September 2021 and closed in March 2022. At the time of study launch, Adventist churches in the UK were under nationwide closures due to the COVID-19 pandemic, therefore the survey was disseminated online, and it also meant that the study used a convenience sampling method, such that any Adventist who wished to participate by filling out the online survey were allowed to do so. The survey was also promoted on social media sites to non-Adventists, who served as controls.

The minimum sample size required for each group of Adventists and non-Adventists was determined to be 38, which allowed for the detection of clinically significant difference in the risk of COVID-19 infection between Adventists and non-Adventists with 80% power at the level of significance of 5%. This was based on the results of a systematic review which suggested that participants following healthy dietary patterns such as the Mediterranean diet, healthy plant-based diets, or the DASH (Dietary Approaches to Stop Hypertension) diet had an up to 77% decreased risk of COVID-19 infection (Sharma et al., 2023). Since Adventists are also encouraged to eat a healthy diet, it was assumed that the effects observed for the healthy diets in the systematic review could reasonably be expected in the Adventist population as well; therefore, the results of this systematic review were deemed appropriate for the power calculation. The sample size was calculated using the below formula taken from Sakpal (2010), which is suitable for comparing two proportions:

$${\text{n }} = \, \left[ {\left( {{\text{Z}}\alpha /{2} + {\text{ Z}}\beta } \right){2} \times \, \left\{ {({\text{p1 }}\left( {{1} - {\text{p1}}} \right) \, + \, \left( {{\text{p2 }}\left( {{1} - {\text{p2}}} \right)} \right)} \right\}} \right]/\left( {{\text{p1 }} - {\text{ p2}}} \right){2}$$

In this formula, Zα/2 (1.96) and Zβ (0.85) are critical values related to the statistical power and significance level used, whereas p1 represents the estimated proportion of COVID-19 infection in Adventists, and p2 is the same risk among non-Adventists. The estimated COVID-19 infection rate for non-Adventists was based on the results of a cohort study of UK healthcare workers who had a COVID-19 infection prevalence of 31.6% (Grant et al., 2020).

The questionnaire was created electronically using the website onlinesurveys.co.uk. The survey included a food frequency questionnaire (FFQ) and a 24-h recall. Additionally, participants were also asked to provide information regarding their general medical history and lifestyle through a series of questions. The survey link was disseminated on various social media platforms, including Facebook groups and Instagram. In addition, a sample of non-Adventists was recruited to serve as a comparison group. The follow-up survey was sent to consenting participants via email in February 2024.

### Questionnaire Development

The food frequency questionnaire utilised in this research was adapted from the validated FFQ, which was used in the EPIC-Norfolk Study (Epic-Norfolk (no date)) since participants in this study were also from the UK and were asked questions regarding the frequency of consumption of various animal- and plant-based products. The food frequency questionnaire of the survey consisted of three sections. The first section included common food items, whereas the second part of the FFQ measured the consumption of meat and meat alternatives. The final section assessed the frequency of nutritional supplement use as well as the consumption of alcoholic and non-alcoholic beverages.

Moreover, participants’ habits and behaviours during the COVID-19 pandemic were also evaluated by utilising a questionnaire developed by Visser et al. (2020), which aimed to assess the impact of the pandemic on participants’ nutritional habits and physical activity.

### Statistical Analysis

All statistical analysis was conducted in SPSS version 29.0.1.1. The Kolmogorov–Smirnov test of normality was used to assess the distribution of continuous variables since this test is more appropriate for samples with over 50 participants than the Shapiro-Wilks test (Mishra et al., 2019). The data were also assessed visually using a histogram. Independent-samples *t*-test was used to compare the mean of a continuous variable between the two categories of a categorical variable. For this, the equality of variances was tested with Levene’s test (Levene, 1960).

Multivariate ANOVA (MANOVA) was used to test whether there were any differences in the mean BMI, WHR and waist circumference values between Adventist and non-Adventist participants.

The Pearson Chi-square test of independence was utilised to test for potential associations between categorical variables with at least 2 categories. The Fisher’s exact test was used if the expected count in any cell was less than 5. Phi was used as an effect size indicator, where 0.1 counted as a small effect, 0.3 as a medium effect and 0.5 as a large effect. For variables with more than 2 categories Cramer’s V was used as an effect size.

Potential linear associations between two continuous variables were investigated using the Pearson correlation. Assumptions such as homogeneity of variances and linearity were checked before conducting the test.

Odds ratios for COVID-19 infection with corresponding 95% confidence intervals were calculated for specific food items with sufficient contrasts in intake from the FFQ data. Vegans, vegetarians and pescatarians were combined into a single variable “plant-based” in some of the statistical analyses. As there is not a uniform definition of a plant-based diet in the scientific literature (Storz, 2021), we used the term to define a diet without the regular use of meat products, as this is consistent with the use of the word in half of the dietary intervention studies that have used it.

COVID-19 disease incidence rates for Adventists and non-Adventists were calculated from the follow-up data collected after 2 years using them following formula and were expressed as a rate per 1000 person-years:

$${\text{Incidence rate}}\, = \,\left( {{\text{number of new cases}}/{\text{person years}}} \right)\, \times \,{1}000$$

Last Observation Carried Forward (LOCF) imputation was used for missing data on the follow-up questionnaire to account for the missing values, which is a method used in longitudinal designs where the last observed non-missing value is used for a participant as the LOCF method assumes that the responses provided are constant. Multinomial logistic regression analysis was used to assess the potential association between COVID-19 symptom severity and plant-based dietary status.

## Results

### Characteristics of Adventist versus Non-Adventist Participants

Participants’ characteristics are summarised in Table 1. In total 170 participants filled in the online survey at baseline. Adventists were significantly older (mean 32.2 years, SD 11.2) than non-Adventists (mean 28.3 years, SD 5.9 (*p* < 0.001)).

**Table 1:** Participant characteristics (n = 170)

Characteristic	Adventists	Non-Adventists
Gender (n)		
Female	63	59
Male	23	25
Age (years)		
Mean Age	36.55	28.34 (p < 0.001)
Standard Deviation	13.34	5.92
Weight (kg)		
Mean Weight	68.12	70.43 (p = 0.69)
Standard Deviation	17.35	17.76
BMI		
Mean BMI	23.68	23.80 (p = 0.69)
Standard Deviation	4.59	5.17
Education Level (n)		
GCSEs or below	8	9
A-levels or equivalent	10	13
Undergraduate degree	32	35
Masters degree	25	20
Doctorate	5	1
Ethnicity (n)		
African Black	4	0
Black Caribbean	19	0
White British	4	1
White European	52	83
White Other	2	0
Asian Other	2	0
Salary (n)		
£0-£29,000	40	64
£29,000-£49,000	26	9
£50,000 +	12	2

Alcohol consumption was strongly correlated with being a non-Adventist (*p* < 0.001). Furthermore, Adventists were also significantly less likely to drink coffee (*p* = 0.001, Phi 0.46) and more likely to fast (*p* = 0.001, Phi 0.34) compared to non-Adventists.

There was a significant association (*p* = 0.02, Phi 0.263) between being an Adventist and the consumption of a plant-based diet; however, following a plant-based diet was not significantly associated with the risk of COVID-19 infection at baseline (OR 0.79, 95% CI 0.41, 1.51). Multivariate ANOVA showed that Adventists’ body mass index (BMI) (23.2, SD 4.6) was not significantly different from that of non-Adventist participants (23.2, SD 5.1) (*p* = 0.99).

### COVID-19 Pandemic

At baseline, 165 respondents provided information about previous COVID-19 infection, of whom 64% stated that they had not been diagnosed with COVID-19 disease, and 36% of respondents stated that they had been diagnosed with the disease prior to completing the survey.

The mean BMI of participants with a COVID-19 diagnosis at baseline was 24.4 (SD 4.7), while the mean BMI of participants who did not have a COVID-19 diagnosis was 23.4 (SD 5.0), but the difference between groups was not significant (*p* = 0.94).

### Impact of Pandemic Measures

Vegetarian Adventists were significantly less likely than non-vegetarian Adventists to increase their weight during the pandemic (OR 0.38, 95% CI 0.17, 0.85). Calorie intake did not seem to impact Adventists and non-Adventists differently (*p* = 0.97) as most (*n* = 130) participants reported no change in calorie intake during the pandemic. Only 30 participants stated that they consumed more calories because of the pandemic; however, they were evenly distributed between the two groups.

In general, regular meat eaters in the whole sample were 43% less likely to maintain or lose weight during the pandemic (OR 0.57, 95% CI 0.42, 0.78).

The impact of the pandemic measures on participants mental health and daily lives was assessed. Four and a half per cent of respondents confirmed a diagnosis of anxiety or depression after the start of the COVID-19 pandemic, and 47.1% of respondents stated that they had experienced more anxiety during the pandemic than before. Furthermore, 37.3% of participants confirmed that they had felt more depressed after the start of the pandemic than before. Fifty per cent of all respondents answered “yes” to the question “Do you use meditation as a stress management technique”, and 47% of participants reported to have used prayer for the same purpose; however, all of the Adventists selected prayer as a stress management technique. Furthermore, there was a significant association between being an Adventist and practising praying for stress relief (*p* < 0.001, Phi 0.61). Singing (*p* < 0.001, Phi 0.32) and reading (*p* < 0.001, Phi 0.741) the Bible as stress management practices were also associated with being an Adventist. Other commonly used stress management techniques were exercise, which was practised by 87% of those responding to this question, and talking to someone, which was utilised by 89% of respondents.

Adventists were 60% more likely to experience less anxiety or no change in anxiety than non-Adventists (OR 1.60, 95% CI 1.16, 2.21), meaning they were less likely to experience anxiety due to the pandemic. Logistic regression confirmed that age was negatively associated with anxiety experienced during the pandemic such that for each one-year increase in age, there was a 5.4% decrease in the odds of experiencing more anxiety (OR 0.946, 95% CI 0.912–0.982). Females were almost three times as likely to experience more anxiety in the same model (OR 2.96, 95% CI 1.38–6.37).

With regard to food availability, 41% of participants reported that they sometimes faced increased difficulties in obtaining groceries during the pandemic, and this was not influenced by socioeconomic status when comparing those with an annual household income of below £29,000 to those with higher income (*p* = 0.34).

### Two-Year Follow-Up

The follow-up survey was sent to all consenting participants via email and contained 11 questions. The questionnaire was filled in by 154 out of the 170 individuals who completed the baseline questionnaire, which means 16 were lost to follow-up. Of those participants who did not respond to the follow-up survey, 4 were Adventists and 12 were non-Adventists, which means data were available for 82 Adventists and 72 non-Adventists in total.

#### Infection Incidence

Eleven of the Adventists and 19 of the non-Adventists reported having had a COVID-19 infection during the observational period (OR 0.45, 95% CI 0.2, 1.0, *p* = 0.05). This meant that the disease incidence rate among Adventists was 65.48 per 1000 person-years and 121.79 per 1000 person-years among the non-Adventists.

Ten participants (33.3%) were plant-based, and twenty (66.7%) were meat eaters of those who reported a COVID-19 infection during follow-up.

#### Symptom Severity

A borderline statistically significant association was shown between vegetarian status and symptom severity by multinomial logistic regression with a Chi-square of 5.828 (2 df, *n* = 30, *p* = 0.05). Nagelkerke’s R-squared value showed that 19.9% of the variance in COVID-19 symptom severity could be explained by plant-based or omnivore status. However, the odds ratios were non-significant for vegetarian status and mild (Exp(B) = 0.222, 95% CI 0.02, 2.42) or moderate symptoms (Exp(B) = 3.33, 95% CI 0.515 to 21.584) compared to severe symptoms in comparison with meat eaters.

#### Long COVID Symptoms

Participants were also asked whether they experienced any persistent symptoms of COVID-19 following infection. Eight Adventists and twenty non-Adventists reported suffering from symptoms that persisted and remained for a longer period after the initial infection, a difference which was significant between the groups (OR 0.30, 95% CI 0.12, 0.78, *p* = 0.01). The most common of these persistent and longer lasting symptoms of COVID-19 were shortness of breath, pain in the chest, difficulty breathing, decreased performance in the gym, and less energy.

## Discussion

The follow-up questionnaire sent out at 2 years revealed that Adventists had a lower COVID-19 incidence and fewer Adventists had persisting symptoms of COVID-19, also known as long COVID. The study showed that two of the most frequently reported symptoms of long COVID were fatigue and breathing difficulties. This finding agrees with the results of the study conducted by Kim et al. (2023), who surveyed 132 participants and showed that the most common long COVID symptom experienced by participants was fatigue (34.8%).

At follow-up, Adventists had a lower infection incidence and vegetarians had a borderline statistically significantly lower risk of experiencing severe symptoms of COVID-19. This confirms previous findings such as the study published by (Kim et al., 2021) who showed that eating a plant-based diet was associated with 73% lower odds of experiencing moderate to severe symptoms of COVID-19 infection. Plant-based diets have been shown to reduce the levels of pro-inflammatory cytokines, which may explain the potential protective effects of plant-based diets concerning COVID-19 severity (Storz, 2021).

This present study found that Adventists experienced less anxiety during the COVID-19 pandemic. Although not in the context of COVID-19, but Beezhold et al. (2010) investigated the relationship between mood states among vegetarian and non-vegetarian Adventists and it showed that vegetarian Adventists were significantly less likely to report negative emotions than omnivores, which suggest that the plant-based diet followed by Adventists has an important role in their emotional well-being.

Furthermore, the practise of prayer was found to be a prominent stress management technique and it was widely practised among the participants, which may have contributed to the lower anxiety experienced by Adventists during the pandemic. There have been studies investigating the potential effects of prayer on anxiety, with some studies, suggesting a reduction in anxiety symptoms among individuals who prayed on a regular basis (Shultz, 2015). Therefore, it is possible that the combination of the Adventist lifestyle, with its emphasis on vegetarianism and prayer, may have worked synergistically to alleviate anxiety in the face of the COVID-19 pandemic. It is worth considering that these findings expand our knowledge of the impact of the Adventist lifestyle on anxiety by suggesting that in addition to diet and lifestyle, prayer as a stress management technique alleviates anxiety symptoms and these findings may have implications beyond the context of the COVID-19 pandemic. In addition to a healthy diet and prayer, a strong social support network may also be a key factor in a pandemic.

In this regard, it may be somewhat of a surprise, but this study found that the spiritual life of Adventists was not impacted negatively by the church closures. A potential explanation for this phenomenon is that churches utilised digital media platforms for sermons, bible studies and regular meetings to help church members stay connected and engaged as suggested by a study conducted in Poland. That particular study showed that the pandemic led to a notable increase in media production and in use of some digital media formats produced by the church. These efforts were implemented in response to the challenging social circumstances created by the pandemic and in response to discriminatory laws that restricted the ability of churches to congregate (Kołodziejska, 2021).

### Strengths and Limitations

Firstly, a noteworthy strength of the study is the determination of sample size using a power calculation, which increases its statistical power for establishing a difference in risk of COVID-19 infection among Adventists and non-Adventists.

The study revealed a number of meaningful differences between the Adventists and non-Adventists even in the context of the COVID-19 pandemic, which may offer useful insights into the impact diet, and lifestyle may have on people’s ability to cope with a global health crisis. The study confirms Adventists’ tendency towards eating a plant-based diet, as well as their abstention from alcohol and coffee, and the practice of fasting, all of which are in agreements with the findings of other cohort studies involving Adventists living in other geographical areas.

An important limitation of this study is that the online survey was self-administered by the participants, which could potentially introduce measurement error for self-reported anthropometric measures such as waist or hip circumference (Bauhoff, 2014).

## Conclusion

Adventists were significantly older in this sample than non-Adventists, yet they had a lower incidence of self-reported COVID-19. This study showed that the highest consumers of red meat and alcohol had a higher risk of COVID-19, which should be further explored in future research as these findings could inform public health promotion strategies that focus on infectious disease prevention through diet and lifestyle.
